# Detection of a novel Shamonda *Orthobunyavirus* in dairy cattle, France, June 2026

**DOI:** 10.2807/1560-7917.ES.2026.31.33.2600689

**Published:** 2026-08-20

**Authors:** Mikail Kelleci, Maxime Fusade-Boyer, Delphine Chrétien, Elodie Durand, Mathias Mircovich, Aurélie Sécula, Benjamin Linard, Nicolas Herman, François Schelcher, Guillaume Croville, Stephan Zientara, Pierre Bessière, Jean-Luc Guérin

**Affiliations:** 1 Université de Toulouse, École Nationale Vétérinaire de Toulouse (ENVT), Institut national de recherche pour l'agriculture, l'alimentation et l'environnement (INRAE), Interactions Hôtes-Agents Pathogènes (IHAP), Toulouse, France; 2 Université de Toulouse, Institut national de recherche pour l'agriculture, l'alimentation et l'environnement (INRAE), Mathématiques et Informatique Appliquées de Toulouse (MIAT), Castanet-Tolosan, France; 3 Clinique vétérinaire des Mazets, Rioms-ès-Montagnes, France; 4 UMR Virologie, Institut national de recherche pour l'agriculture, l'alimentation et l'environnement (INRAE), École Nationale Vétérinaire d’Alfort, ANSES Laboratoire de Santé Animale, Maisons-Alfort, France

**Keywords:** orthobunyavirus, simbu, shamonda, cattle, emergence

## Abstract

In June 2026, acute fever, diarrhoea, lethargy and marked reduction of milk yield were reported in dairy cattle in eastern France. Unbiased Nanopore metagenomics on pooled plasma from affected cows detected Simbu serogroup *Orthobunyavirus,* provisionally named European Shamonda Virus, and recovered complete genomes. Segments L and M clustered with Nigerian Shamonda virus, whereas S showed a distinct clustering pattern, suggesting high mutation rate or reassortment. Similar findings in neighbouring countries indicate cross-border emergence requiring coordinated surveillance.

Orthobunyaviruses are a large genus of arboviruses able to cause disease in humans and animals [1]. One of the largest serogroups of orthobunyaviruses is the Simbu serogroup. Here we describe detection of a novel European Shamonda virus (ESV) of Simbu serogroup associated with acute illness in dairy cattle.

## Current event

In June 2026, veterinarians started reporting clusters of acute diarrhoea, transient fever, lethargy and marked but reversible reductions in milk yield in dairy cattle in the departments of Doubs and Ain, then, in July, in Savoie and Haute-Savoie, in eastern France. Similar clinical episodes were reported first informally in general media and the dairy industry at the same time, and later confirmed in Germany, Switzerland and the Netherlands [2-4]. Initial investigations considered heat stress, nutritional or metabolic disorders and known infectious causes. Schmallenberg virus (SBV) specific RT-qPCR tests performed during the early investigations were negative. We aimed to identify a potential infectious agent, provide its initial genomic characterisation and investigate its potential pathological impact.

## Field investigation and metagenomic detection

Across the nine dairy herds investigated initially in eastern France, the clinical episode was characterised by a 15–75% decrease in herd bulk milk production, associated with diarrhoea, depression or anorexia and, less consistently, fever. The proportion of cows with diarrhoea ranged from 8 to 100% between herds (number of cows on each farm: 25–275), and diarrhoeic episodes generally lasted 1–6 days. Detailed herd-level epidemiological and clinical data are provided in Supplementary Table 4.

An initial unbiased metagenomic analysis [5] of faecal samples collected from diarrhoeic cows did not identify a plausible causative agent. Plasma and faecal samples were subsequently collected from 14 symptomatic dairy cows from three affected farms in Haute-Savoie. All sampled animals had developed fever and/or depression within the preceding 48 h and were still symptomatic at the time of sampling, corresponding to the acute phase of the clinical episode. Samples were combined into three plasma and three faecal pools.

Total RNA was extracted and reverse-transcribed, and sequence-independent cDNA amplification was performed using the SMART-9N method, without requiring prior knowledge of the viral genomes [5]. The amplified products were then used to prepare the sequencing library with the Native Barcoding Kit 96 V14 (Oxford Nanopore Technologies, Oxford, the United Kingdom (UK)) and subsequently sequenced on an Oxford Nanopore Technologies PromethION 2 Solo instrument equipped with a PromethION FLO-PRO114M flow cell (Oxford Nanopore Technologies).

Nanopore metagenomic reads were quality-filtered (fastplong version 0.4.1), host-depleted by mapping to the *Bos taurus* genome with minimap2 version 2.30-r1287 (https://github.com/lh3/minimap2/releases) and taxonomically classified using Kraken2 version 2.1.5 (https://github.com/DerrickWood/kraken2) and KrakenUniq version 1.0.4 (https://github.com/fbreitwieser/krakenuniq). *Orthobunyavirus* reads were detected in all three plasma pools (ca 4,700, 43,500 and 7,500 reads) and at lower abundance in the faecal pools (< 1,000 reads). Reference-guided assembly (minimap2, samtools version 1.23.1 https://www.htslib.org/) and Medaka version 2.0.1 (https://github.com/nanoporetech/medaka) recovered complete L and M coding sequences and a 775-nt partial S segment, deposited in GenBank (PZ797891–PZ797893). The same metagenomic workflow was subsequently applied to samples from five additional affected herds located in three different French regions (Bourgogne-Franche-Comté, Grand Est and Normandie), using plasma of cows or brain tissue from aborted fetuses. In all samples analysed, the Simbu serogroup *Orthobunyavirus* provisionally named European Shamonda Virus (ESV), was the only plausible pathogenic agent detected, enabling us to obtain two new complete genomes, deposited in GenBank (OZ516531–OZ516536). More detailed evidence is presented in Supplementary Table 5.

To assess the initial territorial extent of ESV circulation in dairy farms, plasma samples collected from acutely affected cows in 33 herds (one or two cows per herd) between 28 July and 11 August, and samples from aborted fetuses originating from 18 additional herds (one fetus per herd) sampled between 27 July and 11 August were tested using both a pan-Simbu RT-qPCR assay [6] and an in-house ESV-specific RT-qPCR assay using the primers ESV-F (5′-GCACAGGATTTTGCCCAAGG-3′) and ESV-R (5′-ATTCTTCGCAGCCCCATGAA-3′). In all 18 herds, in which abortions were investigated, an episode of fever and depression in cows had been reported 2–4 weeks before the occurrence of abortions. We detected ESV systematically in all tested samples with both assays. In samples from abortions, viral RNA was consistently detected in the placenta and/or fetal organs, including spleen and brain; brain tissue tested positive in six fetuses, supporting systemic circulation of this virus and raising the possibility of fetal infection associated with abortion. Detailed detection data are presented in Supplementary Tables 6 and 7.

No formal analytical epidemiological study was performed at this stage. However, the consistent detection of novel Shamonda virus RNA in acutely affected cattle from multiple herds, together with the absence of any other plausible infectious agent by untargeted metagenomic analysis, strongly supports ESV as the likely aetiological agent of the observed clinical signs.

## Genomic and phylogenetic characterisation

Maximum-likelihood phylogenetic analyses were performed separately for the three genomic segments using IQ-TREE version 1.6.12 (https://iqtree.github.io/). The best-fitting models were TPM2 + F + R2 for S and GTR + F + I + G4 for L and M. Branch support was assessed using 10,000 ultrafast bootstrap replicates.

Phylogenetic analyses of sequences downloaded from GenBank showed that the virus detected in France belonged to a distinct Shamonda lineage. Based on the L and M segments, the recent French and German viruses clustered with the historical Nigerian Shamonda virus strain Ib An 5550, isolated from cattle in 1965 [7], and were clearly distinct from the lineage comprising Asian and South African Shamonda viruses detected between 2002 and 2023 (Figure). The French virus shared 93.02% and 92.63% nt identity with strain Ib An 5550 for the L and M segments, respectively. French and German viruses shared 99.93% nt identity in both the L and M genome segments. Pairwise nucleotide and amino acid identities with representative members of the Simbu serogroup are provided in Supplementary Tables 1–3.

**Figure:**
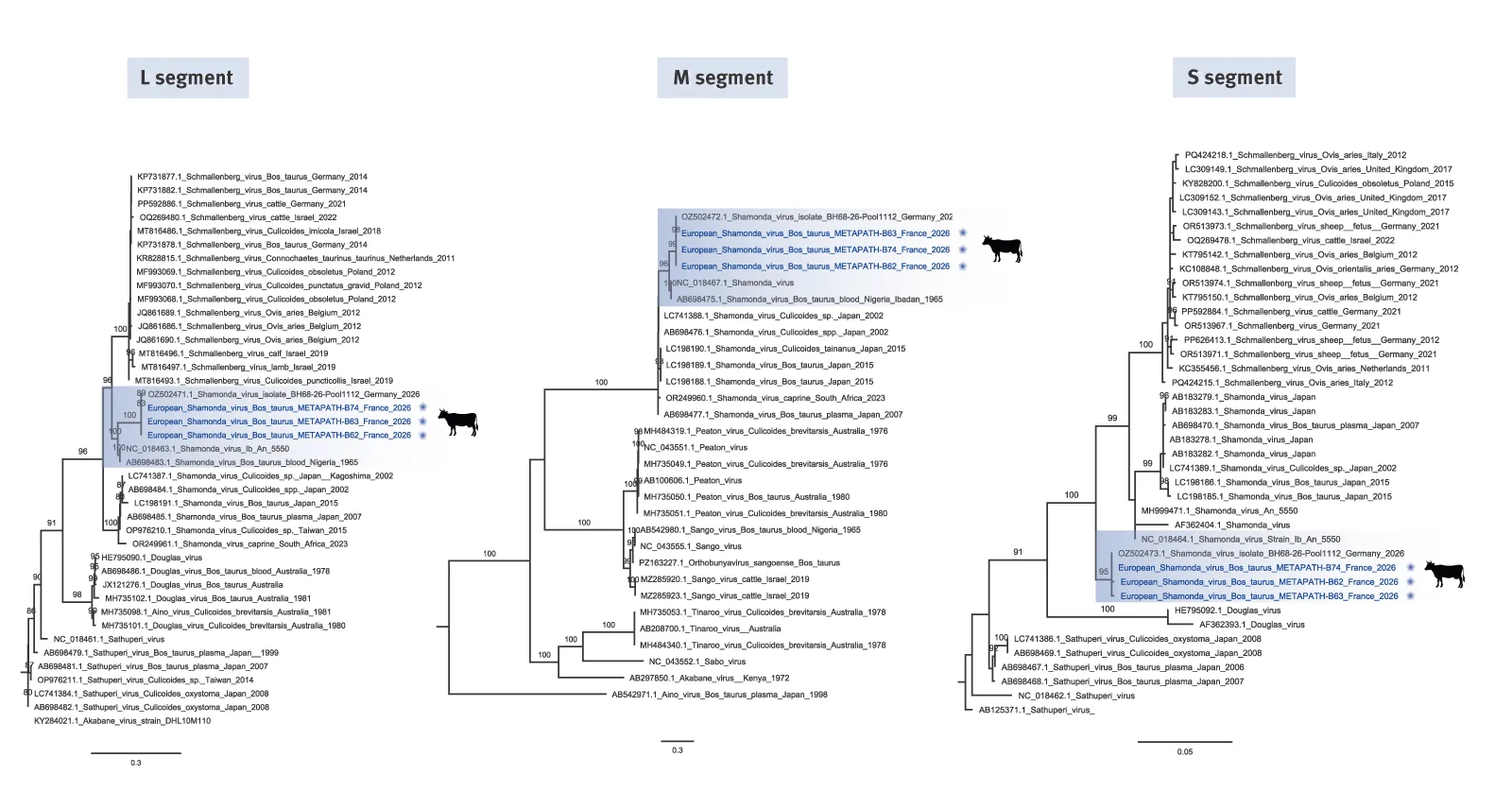
Maximum-likelihood phylogenetic trees of the L, M and S genome segments of the novel European Shamonda virus detected in diseased cattle, France, Germany, and representative Simbu serogroup orthobunyaviruses, 1965–2026

Branch support was assessed using 10,000 ultrafast bootstrap replicates, and only bootstrap values ≥ 90% are shown. The L, M and S trees included 41, 30 and 40 nucleotide sequences, respectively. French strains are labelled in blue with a star, while the blue box highlights the French and German strains and their closest known related strain. For the L and S segment trees, strain DHL10M110 of Akaban virus was used as the outgroup to root the trees (KY284021.1 and KY284023.1) and the sequence of Oropouche from 1991 (AF441119.1) for the M segment. Sequences used as outgroup were omitted from the final figure for clarity. The scale bar indicates the number of nt substitutions per site.

The sequences were downloaded from GenBank.

Phylogenetic analysis showed that the S segments of the French and German viruses, which shared 100% nt identity, did not cluster directly with those of the Nigerian Shamonda virus strains. This phylogenetic observation could be explained by a high mutation rate or a reassortment event involving the S segment. However, if such a reassortment event occurred, the relatively high nucleotide identity observed with Ib An 5550 strain for the S segment (97.1%) suggests that the putative donor virus was likely a genetically closely related member of the Simbu serogroup. No evidence of intrasegment recombination was detected in the S segment using genetic algorithm for recombination detection [8]. The available data do not allow the timing, direction, or the lineage in which such a reassortment may have occurred to be determined.

Overall, the phylogenetic topologies, branch lengths and nucleotide divergence across the three genomic segments support the existence of a distinct Shamonda lineage that has evolved independently from the Asian and South African lineages currently represented in sequence databases and is more closely related to the historical Nigerian Ib An 5550 strain. These findings are consistent with the long-term circulation and independent evolution of this lineage within a largely unsampled viral population, followed by either recent emergence or introduction into France, Germany and Switzerland. The phylogenetic incongruence observed for the S segment further suggests that reassortment may have contributed to the evolutionary history of this lineage.

## Discussion

Orthobunyaviruses are enveloped, negative-sense RNA viruses with three genome segments: L encodes the RNA-dependent RNA polymerase, M encodes the surface glycoproteins Gn and Gc, and S encodes the nucleoprotein and the non-structural protein NSs [1]. Several Simbu serogroup viruses are transmitted by *Culicoides* biting midges and cause disease in ruminants. The M segment is generally the most variable, while L and S are more conserved [9,10].

Shamonda virus was first isolated from cattle in Nigeria in 1965 and was subsequently detected in cattle and *Culicoides* in Japan [7,11]. It has been associated with congenital malformations in calves and was detected in an aborted goat fetus in South Africa in 2023 [11,12]. The complete L protein of the French virus shared 98.5% amino acid identity with Shamonda virus and 97.5% with Schmallenberg virus, data available in Supplementary Table 1. Both values exceed the 96% species demarcation threshold currently used for the genus *Orthobunyavirus*, supporting assignment of the French virus to the species *Orthobunyavirus schmallenbergense*. Nevertheless, its distinct phylogenetic placement and the nucleotide divergence observed across all three genomic segments support its description as a novel Shamonda lineage within this species. To our knowledge, the contemporaneous detections in France and Germany represent the first reported molecular identification of a Shamonda *Orthobunyavirus* in Europe.

The clinical presentation resembles the acute syndrome that accompanied the emergence of Schmallenberg virus in European dairy cattle in 2011 [13,14]. The closely related French and German viral sequences, together with contemporaneous reports of similar disease in Switzerland, then in the Netherlands [2-4], support the emergence of a geographically widespread lineage rather than an isolated event. Its route and timing of introduction into Europe remain unknown. The seasonal occurrence of cases is compatible with vector-borne transmission, but entomological investigations are required to identify the vectors involved and define the geographical extent of circulation.

Priority investigations include individual testing of acute cases, virus isolation, paired serology, retrospective screening of archived samples and surveillance of *Culicoides*. Reproductive surveillance should include abortions, stillbirths and congenital malformations, together with perinatal sampling of dams and offspring. Paired serology and molecular or antigen detection in maternal, placental and fetal/neonatal samples, combined with pathological examination, will be important to assess transplacental infection and reproductive consequences. Experimental infections may subsequently be required to establish causality and determine the duration of viraemia, tissue tropism, transmissibility and reproductive consequences. This event also illustrates the value of untargeted metagenomic sequencing when routine assays directed against known pathogens, including Schmallenberg virus, fail to identify the cause of an emerging syndrome.

*Culicoides*-borne orthobunyaviruses also represent a public health concern. Oropouche virus has caused large outbreaks of febrile illness in humans [15], while Shuni virus, another member of the Simbu serogroup, has been detected in human neurological cases [16]. The Shamonda virus described here is phylogenetically related to these viruses within the genus *Orthobunyavirus*, but no evidence currently indicates that it can infect humans. Its zoonotic potential should therefore be assessed without presuming human pathogenicity.

## Conclusion

Untargeted Nanopore metagenomics identified a novel, European Shamonda virus (ESV) during an acute febrile–diarrhoeic syndrome in French dairy cattle. Discordant phylogenetic placement of the S segment relative to L and M might suggest a reassortment event. Coordinated veterinary, laboratory and public health sector surveillance is now required to define the distribution, clinical impact and reproductive consequences of the virus.

## Data Availability

The viral sequences generated in this study are available in GenBank under accession numbers: PZ797891–PZ797893; OZ516531–OZ516536.

## Supplementary Data

1075000 Supplementary Material
