# Supplementary material for: Detection of a novel Shamonda Orthobunyavirus in dairy cattle, France, June 2026
Source: Euro Surveill. 2026 Aug 20;31(33):2600689. doi: 10.2807/1560-7917.ES.2026.31.33.2600689 (PMC13498902; doi:10.2807/1560-7917.ES.2026.31.33.2600689)
Supplement: Supplementary file 1 [file 26-00689_GUERIN_Supplement.pdf]

Title: Detection of a novel Shamonda Orthobunyavirus in dairy cattle, France, June 2026.

This supplementary material is hosted by *Eurosurveillance* as supporting information alongside the article **Detection of a novel Shamonda Orthobunyavirus in dairy cattle, France, June 2026**, on behalf of the authors, who remain responsible for the accuracy and appropriateness of the content. The same standards for ethics, copyright, attributions and permissions as for the article apply. Supplements are not edited by *Eurosurveillance* and the journal is not responsible for the maintenance of any links or email addresses provided therein.

Supplementary Table S1: Nucleotide and amino acid sequence identity values for the L segment (RNA-dependent RNA polymerase).

Percent nucleotide or amino acid sequence identity.

	**ESV**[a]	SHAV	SATV	DOUV	SBV	AINOV	AKAV	SIMV	PEAV	SHUV	SANV
**ESV**[a]		**98.5**	**95.2**	**94.3**	**97.5**	**68.7**	**70.9**	**69.7**	**68.4**	**68.5**	**69.1**
SHAV	**93.0**		95.8	94.5	98.4	68.8	70.9	69.8	68.5	68.5	69.2
SATV	**84.4**	84.9		96.2	95.9	68.4	71.0	70.4	68.4	68.5	68.8
DOUV	**84.3**	84.6	87.1		94.9	68.7	70.5	70.2	68.6	68.5	68.9
SBV	**89.3**	93.0	84.5	84.6		69.0	71.0	70.0	69.0	68.7	69.4
AINOV	**66.6**	66.0	66.4	66.4	66.8		68.2	71.3	88.7	95.2	90.2
AKAV	**67.2**	67.1	67.4	67.1	67.4	66.4		70.2	68.5	68.2	68.3
SIMV	**67.2**	67.3	67.5	67.7	67.7	68.2	67.5		71.0	71.2	71.1
PEAV	**66.0**	65.9	66.4	66.3	67.0	78.4	66.0	67.6		88.9	93.9
SHUV	**66.0**	66.2	65.8	66.6	66.1	85.0	65.7	67.9	77.9		90.5
SANV	**66.6**	66.7	66.8	66.3	66.9	79.2	65.9	68.3	84.8	78.7	

Values in the bottom left half of the data field represent nucleotide sequence identity, and values in the top right half represent amino acid sequence identity.

Identities were calculated on the coding sequence, from independent pairwise global alignments (Needleman-Wunsch); nucleotide alignments were guided by the protein alignment so that codons are preserved. The same codon-resolved mask is used for NT and AA: a codon is counted only when both triplets and both amino acids are resolved, so every NT denominator is exactly three times the corresponding AA denominator.

GenBank accession numbers: SHAV (Shamonda virus, NC_018463), SATV (Sathuperi virus, NC_018461), DOUV (Douglas virus, HE795090), SBV (Schmallenberg virus, NC_043583), AINOV (Aino virus, NC_018465), AKAV (Akabane virus, NC_009894), SIMV (Simbu virus, NC_018476), PEAV (Peaton virus, NC_043550), SHUV (Shuni virus, NC_043699), SANV (Sango virus, NC_043556).

Values in bold indicate identities involving ESV.

[a] ESV, European Shamonda-like virus: this study, novel Shamonda-like orthobunyavirus consensus sequence (barcode74, consensus v2), August 2026; pair-specific denominators are listed in identites.tsv and use the common codon mask (NT = 3 × AA).

Supplementary Table S2: Nucleotide and amino acid sequence identity values for the M segment (Gn-NSm-Gc polyprotein).

Percent nucleotide or amino acid sequence identity.

	**ESV[a]**	SHAV	SATV	DOUV	SBV	AINOV	AKAV	SIMV	PEAV	SHUV	SANV
**ESV[a]**		**97.6**	**39.4**	**39.7**	**39.0**	**38.5**	**49.2**	**41.4**	**48.9**	**39.5**	**49.4**
SHAV	**92.6**		39.3	39.4	39.2	38.5	49.4	41.4	49.1	39.0	49.6
SATV	**50.4**	50.3		91.6	90.2	58.3	39.2	51.5	41.8	57.1	41.0
DOUV	**50.4**	50.2	85.9		89.0	58.4	39.4	51.5	41.3	57.8	40.5
SBV	**49.7**	49.6	82.2	81.7		58.1	39.4	52.3	41.0	57.3	40.8
AINOV	**49.9**	49.2	60.4	61.0	60.6		39.6	53.8	39.5	75.9	39.6
AKAV	**54.5**	54.5	50.8	50.4	50.4	49.7		41.4	50.4	40.0	49.8
SIMV	**51.8**	51.8	57.5	57.8	57.6	59.5	50.3		41.2	52.2	41.8
PEAV	**55.5**	55.4	51.5	51.5	50.9	50.2	56.3	50.8		39.5	89.9
SHUV	**51.4**	50.6	60.9	60.8	61.2	71.6	50.2	59.3	50.8		40.4
SANV	**55.8**	56.0	51.7	51.8	50.5	50.5	56.7	51.9	83.9	51.7	

Values in the bottom left half of the data field represent nucleotide sequence identity, and values in the top right half represent amino acid sequence identity.

Identities were calculated on the coding sequence, from independent pairwise global alignments (Needleman-Wunsch); nucleotide alignments were guided by the protein alignment so that codons are preserved. The same codon-resolved mask is used for NT and AA: a codon is counted only when both triplets and both amino acids are resolved, so every NT denominator is exactly three times the corresponding AA denominator.

GenBank accession numbers: SHAV (Shamonda virus, NC_018467), SATV (Sathuperi virus, NC_018466), DOUV (Douglas virus, HE795091), SBV (Schmallenberg virus, NC_043584), AINOV (Aino virus, NC_018459), AKAV (Akabane virus, NC_009895), SIMV (Simbu virus, NC_018478), PEAV (Peaton virus, NC_043551), SHUV (Shuni virus, NC_043697), SANV (Sango virus, NC_043555).

Values in bold indicate identities involving ESV.

[a] ESV, European Shamonda-like virus: this study, novel Shamonda-like orthobunyavirus consensus sequence (barcode74, consensus v2), August 2026; pair-specific denominators are listed in identites.tsv and use the common codon mask (NT = 3 × AA).

Supplementary Table S3: Nucleotide and amino acid sequence identity values for the S segment (nucleoprotein N).

Percent nucleotide or amino acid sequence identity.

	**ESV[a]**	SHAV	SATV	DOUV	SBV	AINOV	AKAV	SIMV	PEAV	SHUV	SANV
**ESV[a]**		**99.5**	**100.0**	**99.5**	**99.5**	**80.4**	**80.4**	**79.9**	**81.3**	**80.7**	**81.3**
SHAV	**97.0**		99.6	99.1	100.0	79.8	79.8	80.3	80.7	80.2	80.7
SATV	**94.1**	93.0		99.6	99.6	80.3	80.3	80.3	81.1	80.6	81.1
DOUV	**93.3**	93.0	93.0		99.1	80.3	80.3	80.7	81.1	80.6	81.1
SBV	**95.3**	97.7	93.1	92.3		79.8	79.8	80.3	80.7	80.2	80.7
AINOV	**76.7**	76.7	78.2	78.4	77.0		82.8	83.3	99.1	99.6	99.1
AKAV	**77.6**	77.4	78.7	78.2	77.2	77.4		81.5	82.8	82.3	82.8
SIMV	**77.9**	78.7	79.3	80.5	78.7	78.5	78.8		83.3	83.2	83.3
PEAV	**76.6**	77.5	77.7	77.8	77.2	91.3	76.8	77.8		99.6	100.0
SHUV	**78.0**	77.9	78.3	78.5	77.9	94.0	76.3	78.2	91.7		99.6
SANV	**76.4**	77.4	78.1	78.4	78.2	91.3	77.7	77.5	92.6	91.1	

Values in the bottom left half of the data field represent nucleotide sequence identity, and values in the top right half represent amino acid sequence identity.

Identities were calculated on the coding sequence, from independent pairwise global alignments (Needleman-Wunsch); nucleotide alignments were guided by the protein alignment so that codons are preserved. The same codon-resolved mask is used for NT and AA: a codon is counted only when both triplets and both amino acids are resolved, so every NT denominator is exactly three times the corresponding AA denominator.

GenBank accession numbers: SHAV (Shamonda virus, NC_018464), SATV (Sathuperi virus, NC_018462), DOUV (Douglas virus, HE795092), SBV (Schmallenberg virus, NC_043582), AINOV (Aino virus, NC_018460), AKAV (Akabane virus, NC_009896), SIMV (Simbu virus, NC_018477), PEAV (Peaton virus, NC_043549), SHUV (Shuni virus, NC_043698), SANV (Sango virus, NC_043557).

Values in bold indicate identities involving ESV.

[a] ESV, European Shamonda-like virus: this study, novel Shamonda-like orthobunyavirus consensus sequence (barcode74, consensus v2), August 2026; pair-specific denominators are listed in identites.tsv and use the common codon mask (NT = 3 × AA).

Supplementary Table S4: Epidemiological and clinical data on dairy cattle included in the metagenomic analyses.

Cases	Region (administrative division)	Breeds	Number of lactating cows	Drop in milk production (bulk milk)	Main clinical signs	Percentage of cows with fever (RT > 39.5 °C)	Percentage of cows with depression / anorexia	Percentage of cows with diarrhea	Duration of the bout of diarrhea	Abortion
1	Auvergne-Rhône-Alpes	Montbéliardes	72	40%	Milk drop<br>Diarrhea<br>Depression / anorexia<br>Fever	10%	100% (15 days period)	100%	2–4 days	0
2	Auvergne-Rhône-Alpes	Montbéliardes	80	50%<br>40 to 80% at individual level	Milk drop<br>Diarrhea	0	30%	30%	2–4 days	0
3	Auvergne-Rhône-Alpes	Montbéliardes<br>Abondances	95	50%<br>40 to 80% at individual level	Milk drop<br>Diarrhea	0	8%	8%	4–6 days	0
4	Auvergne-Rhône-Alpes	Montbéliardes<br>Abondances	60	50%<br>50 to 75% at individual level	Milk drop<br>Diarrhea<br>Depression / anorexia	0	20%	20%	1–2 days	0
5	Auvergne-Rhône-Alpes	Montbéliardes	87	25%	Milk drop<br>Diarrhea<br>Depression / anorexia<br>Fever	7%	25%	25%	1–3 days	0
6	Auvergne-Rhône-Alpes	Montbeliardes	150	75%	Milk drop<br>Diarrhea<br>Depression / anorexia<br>Fever	25%	25%	75%	1–3 days	0
7	Auvergne-Rhône-Alpes	Montbeliardes	25	75%	Milk drop<br>Diarrhea<br>Fever	10%	10%	50%	1–3 days	0
8	Grand Est	Prim'holstein	60	20%	Milk drop<br>Diarrhea<br>Depression / anorexia<br>Fever	2%	70%	20%	1–2 days	0
9	Grand Est	Prim'Holstein	275	15%	Milk drop<br>Diarrhea<br>Depression / anorexia<br>Fever	1%	25%	10%	1–2 days	0
10	Auvergne-Rhône-Alpes	Prim'Holstein	125	10%	Milk drop<br>Diarrhea<br>Fever	2%	10%	10%	1–3 days	0

Supplementary Table S4: (continued)

Cases	Region (administrative division)	Breeds	Number of lactating cows	Drop in milk production (bulk milk)	Main clinical signs	Percentage of cows with fever (RT > 39.5 °C)	Percentage of cows with depression / anorexia	Percentage of cows with diarrhea	Duration of the bout of diarrhea	Abortion
11	Normandie	Prim'Holstein	80	ND	Milk drop<br>Diarrhea<br>Fever	ND	ND	ND	2–4 days	0
12	Auvergne-Rhône-Alpes	Montbéliardes	80	25%	Milk drop<br>Diarrhea<br>Depression / anorexia	0	25%	50%	2–4 days	0
13	Bourgogne-Franche-Comté	Montbéliardes	60	ND	Milk drop<br>Diarrhea<br>Depression	0	ND	ND	ND	n=1<br>180–200 days of gestation<br>~3 weeks after milk drop / diarrhea
14	Grand Est	Prim'holstein and Montbéliardes	65	ND	Milk drop<br>Diarrhea<br>Depression	ND	10%	10%	1–3 days	n=2<br>180–200 / >250 days of gestation<br>~3 weeks after milk drop / diarrhea

ND, not determined.

Supplementary Table S5: Detection of European Shamonda virus (ESV) in dairy cattle, France, June 2026.

ESV, a Shamonda orthobunyavirus, detected by untargeted metagenomics with nucleotide and protein evidence.

Animal[1]	Origin	Sample	Ct	Virus[5,6]	Segment	Protein	Reads (nt)[2]	Reads (protein)[3]	Identity to closest reference[4]
26-0601	Auvergne-Rhône-Alpes	Plasma EDTA, pooled	–	ESV	L	RNA-dependent RNA polymerase	27 825	24 014	93.0%
					M	Gn-Gc-NSm polyprotein	13 752	11 917	92.6%
					S	Nucleocapsid protein	732	581	97.4%
26-0741	Auvergne-Rhône-Alpes	Plasma	19.93	ESV	L	RNA-dependent RNA polymerase	391 216	355 567	93.1%
					M	Gn-Gc-NSm polyprotein	116 866	105 875	92.7%
					S	Nucleocapsid protein	9 293	8 434	97.4%
26-0656	Auvergne-Rhône-Alpes	Plasma	22.60	ESV	L	RNA-dependent RNA polymerase	601 623	547 580	93.1%
					M	Gn-Gc-NSm polyprotein	205 374	187 016	92.7%
					S	Nucleocapsid protein	21 725	19 423	97.5%
26-0707	Bourgogne-Franche-Comté	Brain	26.61	ESV	L	RNA-dependent RNA polymerase	2 126	1 928	92.9%
					M	Gn-Gc-NSm polyprotein	2 929	2 662	91.8%
					S	Nucleocapsid protein	361	332	96.8%
26-0768	Grand Est	Brain	27.32	ESV	L	RNA-dependent RNA polymerase	1 546	1 376	93.0%
					M	Gn-Gc-NSm polyprotein	1 269	1 139	92.3%
					S	Nucleocapsid protein	170	61	97.3%
26-0654	Normandie	Plasma, serum	31.68, 32.41	ESV	L	RNA-dependent RNA polymerase	149	104	92.8%
					M	Gn-Gc-NSm polyprotein	66	40	92.2%
					S	Nucleocapsid protein	9	7	97.2%

[1] Six dairy cattle investigated for abortion and milk drop.

[2] Reads assigned by competitive mapping against a 1 736-sequence Peribunyaviridae panel, counted per segment.

[3] Reads whose translation aligns to that protein at 90% amino acid identity or more (DIAMOND blastx against RVDB-prot). A virus is reported only when both layers support it.

[4] Nucleotide identity of the assembled genome to its nearest known relative, by dc-megablast against the whole Peribunyaviridae family (nt_viruses, BLAST+ 2.15.0). That relative is Shamonda virus, strain Ib An 5550 (L NC_018463.1, M NC_018467.1, S NC_018464.1), on all three segments in every sample.

[5] All six samples carry the same virus: L segments 99.75 to 99.95% identical to one another, M segments 98.0 to 99.95%, S segments 99.6 to 99.9%.

[6] No other virus is reported. Every candidate raised by the protein layer proved to be host sequence, bacteriophage or a contaminating fragment.

Supplementary Table S6: Viral RNA detection by RT-qPCR on blood samples from dairy cows.

Viral RNA was extracted from 150 µL of plasma according to the manufacturer's instructions (NucleoSpin RNA virus; Macherey-Nagel). European Shamonda Virus nucleic acid load was determined by RT-qPCR using primers targeting either the S segment (pan-Simbu RT-qPCR) or the M segment (ESV-specific RT-qPCR). RT-qPCR was performed in 96-well plates in a final volume of 20 µL according to the manufacturer's instructions (iTaq SYBR green one-step; Bio-Rad) with 2 µL of RNA and a final concentration of 0.5 µM of each primer.

Submission ID	Sample	Ct values pan-Simbu RT-qPCR	Ct values ESV RT-qPCR	Region (administrative division)	Date of sampling
26-0640	Plasma	24,28	20,99	Auvergne-Rhône-Alpes	30/07/2026
26-0649	Plasma	22,96	19,78	Auvergne-Rhône-Alpes	02/08/2026
26-0655	Plasma	28,12	24,17	Auvergne-Rhône-Alpes	04/08/2026
26-0669	Plasma	30,62	26,23	Auvergne-Rhône-Alpes	04/08/2026
26-0699	Plasma	25,15	21,24	Bourgogne-Franche-Comté	04/08/2026
26-0730	Plasma	34,82	30,35	Auvergne-Rhône-Alpes	05/08/2026
26-0731	Plasma	28,98	37,36	Normandie	05/08/2026
26-0737-1	Plasma	24,44	21,34	Auvergne-Rhône-Alpes	05/08/2026
26-0737-2	Plasma	27,23	23,57	Auvergne-Rhône-Alpes	05/08/2026
26-0738	Plasma	24,26	20,86	Auvergne-Rhône-Alpes	05/08/2026
26-0742	Plasma	23,7	22,06	Auvergne-Rhône-Alpes	05/08/2026
26-0743	Plasma	38,56	36,27	Auvergne-Rhône-Alpes	05/08/2026
26-0744	Plasma	26,39	24,16	Auvergne-Rhône-Alpes	05/08/2026
26-0745	Plasma	24,9	23,2	Auvergne-Rhône-Alpes	05/08/2026
26-0746	Plasma	22,43	20,97	Auvergne-Rhône-Alpes	05/08/2026
26-0748	Plasma	22,44	21,36	Auvergne-Rhône-Alpes	05/08/2026
26-0749	Serum	23,96	22,19	Auvergne-Rhône-Alpes	05/08/2026
26-0750	Serum	23,62	21,95	Auvergne-Rhône-Alpes	05/08/2026
26-0755	Plasma	22,21	20,79	Auvergne-Rhône-Alpes	28/07/2026
26-0757-1	Plasma	26,39	22,96	Grand Est	05/08/2026
26-0757-2	Plasma	36,8	32,3	Grand Est	05/08/2026
26-0758	Plasma	27,34	23,56	Grand Est	04/08/2026
26-0761-1	Plasma	38,61	35,68	Grand Est	04/08/2026
26-0761-2	Plasma	24,22	21,36	Grand Est	04/08/2026
26-0762	Plasma	25,95	22,25	Grand Est	04/08/2026
26-0764	Plasma	27,08	22,66	Grand Est	04/08/2026
26-0836	Plasma	30,55	23,61	Bourgogne-Franche-Comté	10/08/2026
26-0837	Plasma	28,43	21,47	Grand Est	10/08/2026
26-0838	Plasma	29,79	22,49	Sud	10/08/2026
26-0844	Plasma	39,4	30,38	Auvergne-Rhône-Alpes	11/08/2026
26-0845	Plasma	29,48	21,87	Auvergne-Rhône-Alpes	10/08/2026
26-0846	Plasma	28,73	21,84	Auvergne-Rhône-Alpes	11/08/2026
26-0847	Plasma	25,47	18,93	Auvergne-Rhône-Alpes	11/08/2026
26-0850	Plasma	34,86	28,88	Auvergne-Rhône-Alpes	08/08/2026

Supplementary Table S6: (continued)

Submission ID	Sample	Ct values pan-Simbu RT-qPCR	Ct values ESV RT-qPCR	Region (administrative division)	Date of sampling
26-0852	Plasma	27,25	21,5	Auvergne-Rhône-Alpes	10/08/2026
26-0867	Plasma	34,37	28,18	Auvergne-Rhône-Alpes	11/08/2026

Supplementary Table S7: Viral RNA detection by RT-qPCR on placenta and aborted foetuses from dairy cows.

Viral RNA was extracted from approximately 30 mg of tissue according to the manufacturer's instructions (ID Gene Mag Fast; Innovative Diagnostics). European Shamonda Virus nucleic acid load was determined by RT-qPCR using primers targeting either the S segment (pan-Simbu RT-qPCR) or the M segment (ESV-specific RT-qPCR). RT-qPCR was performed in 96-well plates in a final volume of 20 µL according to the manufacturer's instructions (iTaq SYBR green one-step; Bio-Rad) with 2 µL of RNA and a final concentration of 0.5 µM of each primer.

Submission ID	Sample	Ct values pan-Simbu RT-qPCR	Ct values ESV RT-qPCR	Region (administrative division)	Date of sampling
26-0699	Brain	32,9	33,04	Bourgogne-Franche-Comté	04/08/2026
26-0707	Brain	26.61	27,74	Bourgogne-Franche-Comté	04/08/2026
26-0707	Spleen	36.43	35,63	Bourgogne-Franche-Comté	04/08/2026
26-0715	Placenta	36.02	32,27	Bourgogne-Franche-Comté	28/07/2026
26-0716	Placenta	37.72	35,73	Bourgogne-Franche-Comté	28/07/2026
26-0717	Placenta	31.81	29,16	Bourgogne-Franche-Comté	27/07/2026
26-0718	Placenta	35.45	32,67	Bourgogne-Franche-Comté	28/07/2026
26-0719	Placenta	35.07	33,43	Bourgogne-Franche-Comté	27/07/2026
26-0725	Placenta	35.13	32,39	Bourgogne-Franche-Comté	29/07/2026
26-0727	Placenta	34.59	32,6	Bourgogne-Franche-Comté	30/07/2026
26-0735	Abomasal fluid	31.65	29,5	Bourgogne-Franche-Comté	07/08/2026
26-0765	Placenta	38.03	34,04	Bourgogne-Franche-Comté	05/08/2026
26-0768	Brain	27.32	25,55	Grand Est	03/08/2026
26-0788	Placenta	37.08	38,19	Auvergne-Rhône-Alpes	07/08/2026
26-0825	Abomasal fluid	33.10	30,68	Bourgogne-Franche-Comté	05/08/2026
26-0874	Brain	34.32	31,11	Auvergne-Rhône-Alpes	10/08/2026
26-0874	Spleen	37.50	33,54	Auvergne-Rhône-Alpes	10/08/2026
26-0875	Brain	30.58	29,19	Auvergne-Rhône-Alpes	10/08/2026
26-0875	Placenta	32.12	31,3	Auvergne-Rhône-Alpes	10/08/2026
26-0875	Spleen	34.81	34	Auvergne-Rhône-Alpes	10/08/2026
26-0879	Brain	26.93	24,03	Auvergne-Rhône-Alpes	11/08/2026
26-0879	Spleen	36.51	34,07	Auvergne-Rhône-Alpes	11/08/2026
26-0880	Abomasal fluid	30.06	30,41	Auvergne-Rhône-Alpes	11/08/2026
